# An oversampling method for multi-class imbalanced data based on composite weights

**DOI:** 10.1371/journal.pone.0259227

**Published:** 2021-11-12

**Authors:** Mingyang Deng, Yingshi Guo, Chang Wang, Fuwei Wu

**Affiliations:** 1 School of Automobile, Chang’an University, Xi’an, China; 2 College of Automobile Engineering, College of Humanities and Information Changchun University of Technology, Changchun, China; National University of Sciences and Technology, PAKISTAN

## Abstract

To solve the oversampling problem of multi-class small samples and to improve their classification accuracy, we develop an oversampling method based on classification ranking and weight setting. The designed oversampling algorithm sorts the data within each class of dataset according to the distance from original data to the hyperplane. Furthermore, iterative sampling is performed within the class and inter-class sampling is adopted at the boundaries of adjacent classes according to the sampling weight composed of data density and data sorting. Finally, information assignment is performed on all newly generated sampling data. The training and testing experiments of the algorithm are conducted by using the UCI imbalanced datasets, and the established composite metrics are used to evaluate the performance of the proposed algorithm and other algorithms in comprehensive evaluation method. The results show that the proposed algorithm makes the multi-class imbalanced data balanced in terms of quantity, and the newly generated data maintain the distribution characteristics and information properties of the original samples. Moreover, compared with other algorithms such as SMOTE and SVMOM, the proposed algorithm has reached a higher classification accuracy of about 90%. It is concluded that this algorithm has high practicability and general characteristics for imbalanced multi-class samples.

## 1. Introduction

Imbalanced data is one of the important problems to be solved in machine learning and data mining. Imbalance data classification is widely used in data processing in the fields of social surveys, disaster prediction and disease prevention [1–3]. Studies have shown that in the classification process of imbalanced data, the classification hyperplane boundary is shifted to the side of small samples due to the support of large sample size, and then small samples are misclassified leading to low classification accuracy of imbalanced data. In multi-class imbalanced data, the classification hyperplane is affected by the difference of data sizes of multi-class samples, which makes its classification accuracy unable to meet the needs of scientific computing. Therefore, the classification of multi-class imbalanced data has become a key problem in data processing research [4].

Currently, the common methods of imbalanced data sampling mainly include data oversampling, data undersampling and hybrid sampling. Undersampling is the process of reducing data size of large samples to balance data sizes of different kinds of samples, and needs to be improved continuously due to the fact that discarding data from majority class samples may result in the loss of useful information of majority class. Oversampling takes small samples as the object to generate new samples, which needs to be further optimized due to the frequent occurrence of over-fitting. The hybrid sampling combines the above two methods, but it also needs further improvement due to the longer consumption time. The classical algorithms for these three types of sampling methods are shown in Table 1.

**Table 1:** Classification of typical algorithms for imbalanced sampling and representative literature.

Classification	Strategy	Typical literature
Oversampling	K-order approach	Chawla et al. (2002), Han et al. (2005) [7,8]
	Clustering	Sanchez et al. (2013), Nekooeimehr et al. (2016) [9,10]
	Neural networks	Konno et al. (2019) [11]
Undersampling	Clustering	Yen et al. (2009), Tsai et al. (2018) [12,13]
	Integration	Liu et al. (2009), Tahir et al. (2012) [14,15]
Hybrid sampling	Random sampling	Seiffert et al. (2009) [19]
	SMOTE+ENN/TOMEK	Batista et al. (2004) [18]

In 1993, Anand et al. found that small sample data affected the convergence of neural network classification algorithms, and began to study imbalanced data sampling algorithms [5]. In 1995, Vapnik proposed an algorithm called support vector machine, which laid the foundation for the development of classification algorithms for imbalanced data [6]. In the early stages of oversampling algorithm research, Chawla et al. (2002) proposed the Synthetic Minority Over-sampling Technique (SMOTE) sampling method, which randomly generates new samples based on the average distance between the sample and K neighboring samples, and the sampled samples increase the diversity of the data [7]. Subsequently, Han et al. (2005) proposed an improved SMOTE algorithm called Borderline-SMOTE in order to enhance the sampling training of boundary samples [8]. With the application of artificial intelligence in various fields, Sanchez et al. (2013) and Nekooeimehr et al. (2016) successively introduced the idea of clustering and proposed an oversampling algorithm of intra-layer clustering, which enhanced the classification accuracy by improving the boundary data sampling [9,10]. Konno et al. (2019) applied the artificial neural network algorithm to oversampling, and the classification accuracy was greatly improved [11].

Undersampling has two main representative research directions of clustering and integration. The clustering undersampling proposed by Yen et al. (2009) is mainly to sample representative data in each group, and its sampling accuracy is higher than random sampling [12]. In 2018, Tsai et al. refined the clustering undersampling by utilizing group features instead of features of the actual samples to extend the classification range of the algorithm [13]. Moreover, the integrated undersampling algorithm proposed by Liu et al. (2009) and modified by Tahir et al. (2012) is widely applied [14,15]. With the increasing prominence of the multi-class imbalanced data problem, undersampling has also begun to improve toward the classification of multi-class samples, such as a neighborhood-based undersampling method proposed by Vuttipittayamongkol et al. (2020) and a hashing-based undersampling algorithm proposed by Ng et al. (2020) [16,17].

The researches on hybrid algorithms are mainly based on the use of algorithmic superposition. Initially, Batista et al. (2004) proposed a hybrid algorithm of SMOTE+TOMEK and SOMTE+ENN [18]. However, in the early stage, the hybrid algorithms were dominated by the random hybrid sampling algorithm of Seiffert et al. (2009) [19]. Later, the improved SMOTE+ENN algorithm proposed by Xu et al. (2020) became mainstream algorithm for hybrid sampling [20].

For the classification problem of imbalanced data, besides the improvement of the classical algorithms and the proposal of novel algorithms, some ensemble approaches have been proposed by integrating the classical algorithms with novel strategies, such as the three-way decision ensemble [21] and the samples’ selection strategy [22]. In addition, the sampling algorithms for multi-class imbalanced data have been paid more and more attention in recent years. For example, the multiclass radial-based oversampling (MC-RBO) proposed by Krawczyk et al. (2019) [23] and an oversampling technique based on fuzzy representativeness difference proposed by Ren et al. (2020) [24] have attracted much observation. It can be observed that the research focus is expanding towards multi-class imbalanced data.

From the above analysis, it is clear that oversampling is one of the main methods to solve the problem of excessive differences in the number of imbalanced samples. To solve the overfitting phenomenon of the oversampling algorithms, the existing studies have mainly considered the density characteristics of the original sample data in the sampling process to maintain the invariance of the sample characteristics from the spatial attributes. Zhang et al. (2020) pointed out that the oversampling method based on hyperplane and data density as weights improved the subsequent distribution accuracy [25]. However, it only extracts the basic features of two-class samples and does not consider the problem of feature extraction of imbalanced data with more than three classes. Furthermore, when the data size of the sample is too large, the overfitting phenomenon is unavoidable due to the fact that this algorithm does not consider the information characteristics of multi-class imbalanced data.

To solve this problem, the paper proposes a classification oversampling method based on classification ranking and weight setting. The research goal is to generate oversampled instances that can maintain the spatial distribution characteristics and information features of the original samples while balancing the amount of data between multiple classes of samples, so as to enhance the classification accuracy of multi-class imbalanced data while avoiding overfitting. At first, the data are sorted depending on the distance from the sample data to the hyperplane after the data characteristics of multi-class small samples are analyzed. Next, taking the data sorting and distribution density of the samples as the sampling weights, iterative sampling is carried out within the categories and inter-class sampling is performed at the boundaries of adjacent categories to balance the number of different categories and maintain the distribution characteristics of original sample. Then, the new samples generated are assigned by their neighborhood data information to retain the information attributes of the original samples. After training and comparison tests, it is concluded that the proposed algorithm not only enables the imbalanced data to achieve quantitative balance, but also has good classification accuracy.

## 2. Theory and methods

This study aims to use the oversampling method to achieve the equalization of multi-class imbalanced data, which facilitates the later data analysis to explore the information value of minority class samples. Previous studies have shown that existing oversampling is prone to data over-fitting, which is mainly due to the lack of data characteristics of the original samples in the newly generated data [26–29]. Therefore, the classification oversampling algorithm proposed in this study is to extract the distribution characteristics of the original sample, generate new data based on the composite weights composed of data sorting and data density, and assign values to them with their data information, so as to avoid the phenomenon of data over-fitting and to maintain the data characteristics of the original samples.

### 2.1 Classification data sorting theory

Data sorting is the first step to resolve the characteristics of sample data, which reflects the spatial location relationship of sample data. As the boundary between two categories of data in Euclidean space, the hyperplane itself can intuitively reflect the spatial location relationship between two categories of sample data, however, it cannot be directly applied to multi-class samples. Therefore, this study proposes to map the relative positions of each class of data according to the distance of each class of data relative to the hyperplane in the sorting of multi-class samples, so as to solve the problem of spatial relative positions of multi-class sample data.

**Fig 1:**
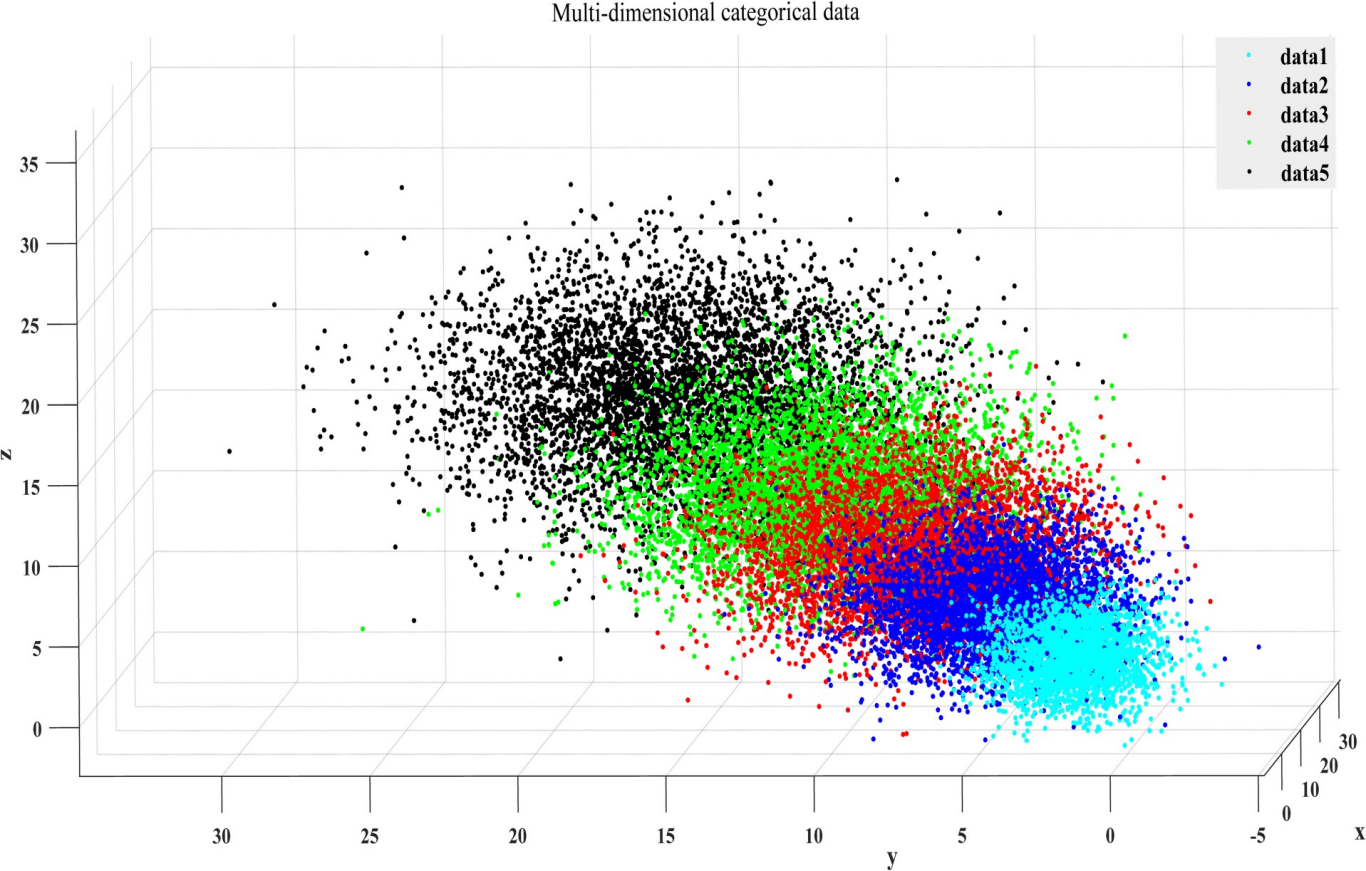
Multi-dimensional classification data.

**Fig 2:**
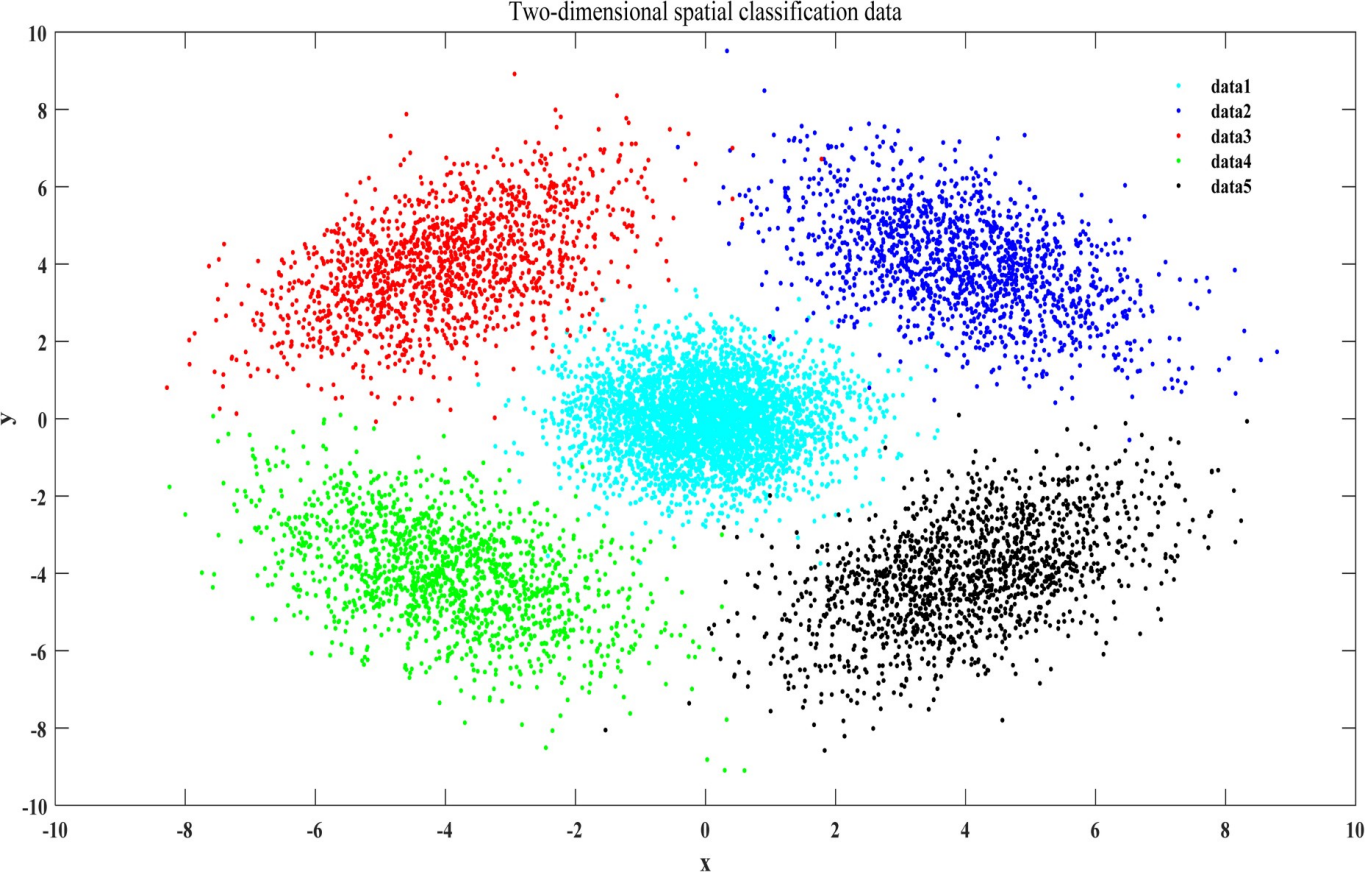
Two-dimensional spatial classification data.

As shown in Fig 1, the hyperplane between each two categories of data is obtained in the three-dimensional space, and the three-dimensional spatial distance from the data to the hyperplane obtained within each class is regarded as the distance feature. Next, the data are sorted by distance, and the three-dimensional spatial data are transformed into two-dimensional spatial data (Fig 2), which will help to solve the problem of data sampling in high-dimensional space while maintaining the distribution characteristics of the original data.

### 2.2 Sorting methods for multi-class imbalanced data

According to the data sorting theory described above, the data boundaries between multi-categorical datasets are obtained by introducing a hyperplane equation [27,30]. Based on this, the sorting of data within each class is realized. The specific solution process of the hyperplane equation is as follows.

#### 2.2.1 Step 1: Hyperplane equations

The hyperplane equation built in this research is:

$$\underset{w,b,\xi }{\min}\frac{1}{2}{\left\|w\right\|}^{2}+c\sum_{i=1}^{n}\xi_{i}$$ (1)

*w* in Eq (1) is the normal vector of the classification surface. Its constraint condition is:

$$y_{i}(w\cdot x_{i}+b)\geq 1-\xi_{i},\;(\xi_{i}\geq 0,i=1,2,\ldots ,n)$$ (2)

The resulting classification hyperplane is:

$$w^{\prime }x+b^{\prime }=0$$ (3)

In order to obtain the optimal solutions *w*′ and *b*′ of the hyperplane equation, the optimal *α*′ obtained is *α*′ = (*α*_1_,*α*_2_,…,*α*_*n*_)^*T*^ through the sequential minimization optimization algorithm [31], and then the optimal solutions w* and b* are derived as follows.


$$w^{\prime }=\sum_{i=1}^{n}\alpha_{i}y_{i}x_{i}$$ (4)

$$b^{\prime }=y_{i}-\sum_{i=1}^{n}\alpha_{i}^{*}y_{i}(x_{i}\cdot x_{j})$$ (5)

#### 2.2.2 Step 2: The distance from data to the hyperplane

For *a* certain class of dataset SD, *x*_*i*_∈*SD*, *Dist*(*x*_*i*_,*D*_*B*) represents the distance from *x*_*i*_ to the decision boundary (*D*_*B*) (see Eq (6)).


$$Dist(x_{i},D\_B)=\frac{\left|w^{T}x+b\right|}{\left\|w\right\|}$$ (6)

#### 2.2.3 Step 3: Sorting results of classification data

Performing steps 1 and 2 for each class of data respectively will obtain the data ordering within each class and the spatial location relationships between the samples of multiple classes.

### 2.3 Data density

The data density is the sum of the Euclidean spatial distances from a single sample data to the surrounding data in the same category sample, to reflect the distribution density of same class data around this sample data. Moreover, the smaller the sum of the distances implies that there are more same class points around the sample data. The result is that the distribution density of this data is greater. The distance from the data point (*x*_*i*_,*y*_*i*_) in Euclidean space to any surrounding point (*x*_*j*_,*y*_*j*_) in the same category sample is $d_{i}=\sqrt{{\left(x_{i}-x_{j}\right)}^{2}+{\left(y_{i}-y_{j}\right)}^{2}}$. To prevent the excessive time complexity of the algorithm, we take the average of the sum of the distances from the sample point to the five nearest neighbor points in its surrounding similar samples as the density feature value of the sample point [32]. That is the 5-point distance average $Density(x_{i})=\frac{1}{5}\sum_{i=1}^{5}d_{i}$ used as the sampling weight.

### 2.4 Assignment of sample information

The data values in the sample represent certain information and are the metric values of physical quantities. There is a certain correlation between the original sample data, and some of the data have certain rules.

Therefore, the reassignment of data information of oversampling new sample should maintain the characteristic rule of the original sample. In this study, weights were set by data density and additional weights of data information, and then oversampling was performed on the samples to avoid the phenomenon of over-fitting. According to the principle of adjacent consistency of the sample data, the data information of the new sample is the average value of the data information of the surrounding neighbors in the same class sample. Furthermore, the information average of *j* neighbors of the new sample data *i* is set to *n*_*i*_, $n_{i}=\frac{1}{m}\sum_{j=1}^{m}n_{j}$, (*m*≤5).

When each class of samples is oversampled, the original sample is sampled according to the weight *S* = *α*⋅*Density*+*β*⋅*Dist*, and *n*_*i*_ is used to assign the data information of the new sample. Finally, we take the value α = β = 0.5 [33].

## 3. Training and application of classification oversampling algorithm

### 3.1 The description and design process for the algorithm

**Fig 3:**
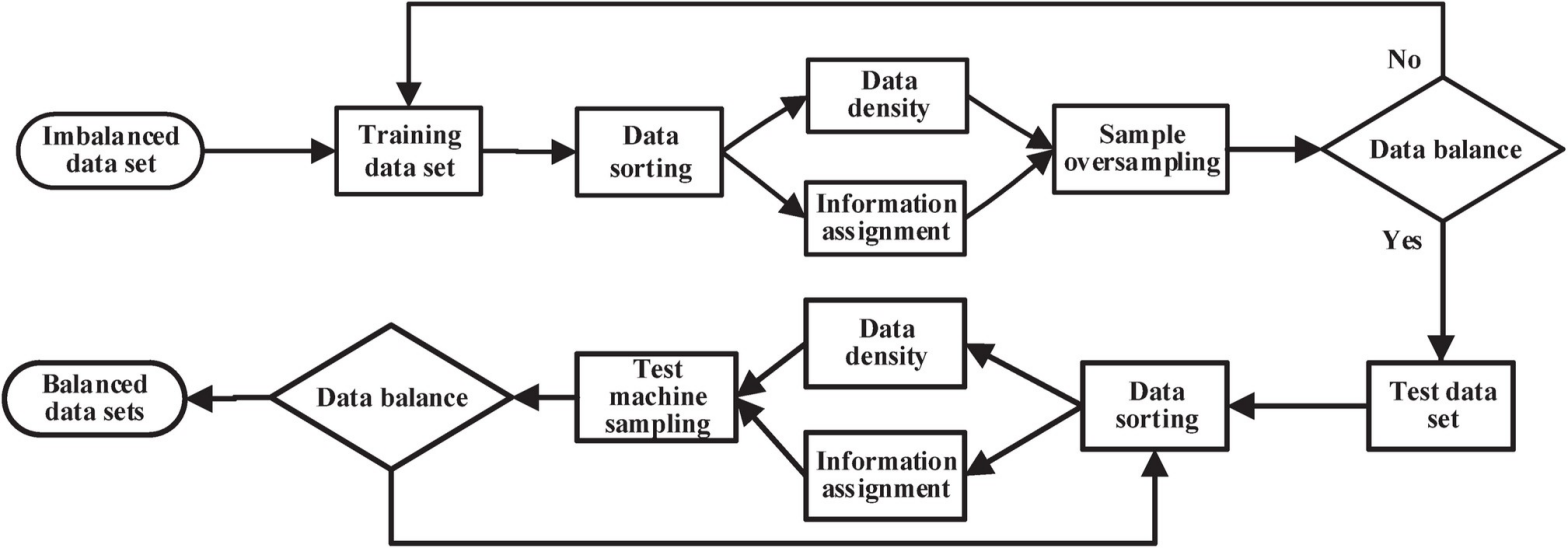
The classification oversampling algorithm flow chart.

The imbalanced dataset is defined as *ID*(1,2,3,…,*N*), where *N* is the number of sample categories, and *M* is the number of small sample categories. In addition, *S* represents the training set, *T* represents the test set, the small sample is represented by *S*_min_ in the training set, and the synthetic sample of the test machine and the small sample *T*_min_ is represented by *S*_*new*_. The algorithm flow of the multi-class imbalanced datasets is shown in Fig 3.

#### 3.1.1 Sampling total

When calculating the total number to be sampled for each class sample, the number of class samples with the largest number is determined as the base number. In addition, the number of minority class samples to be sampled is determined by the difference between the data amount of minority class samples and the selected base number.

#### 3.1.2 Data sorting

According to the distance value $Distance_{x_{i}}$ between the data points and the hyperplane, the sample data are sorted according to the distance from the smallest to the largest, and classification datasets were obtained.

#### 3.1.3 Sample density

When the sample density is solved for each class, the five nearest distance data points of a certain data point in the same sample are selected as the neighbor set, and then the Euclidean spatial distance density of the five neighbor points is solved.

#### 3.1.4 Sample information

The average value of the sample information of the 5 neighboring points is used as the information assignment of the generated data points in the new sample.

#### 3.1.5 Sampling rules and information assignment

The original samples were sampled at two points, three points, and four points in the order of the weight *S* = *α*⋅*Density*+*β*⋅*Dist*, and information was assigned to the generated new sampling points.

#### 3.1.6 End of sampling

Sampling does not terminate until the imbalanced dataset reaches equilibrium.

### 3.2 Algorithm training

To train the proposed classification oversampling algorithm accurately, we selected common imbalanced datasets from the international standard database UCI to train this oversampling algorithm [34]. The selected datasets, such as weather data, clinical cases, financial data, and product sampling, formed the training datasets. The improved algorithm was trained by using MATLAB 2020. Furthermore, market research data as the testing datasets were utilized to compare the sampling accuracy and classification accuracy of different algorithms. Table 2 demonstrates the datasets used to train and test the oversampling algorithm.

**Table 2:** The Datasets for training and testing algorithms.

Datasets	Imbalanced Degree	Minority class	Majority class	Total samples	Usage
Weather data	0.12	163	1358	1521	training
Clinical cases	0.28	6636	23700	30336	training
Financial data	0.31	178	574	752	training
Product sampling	0.56	126	225	351	training
Market Research	0.43	431	10048	10479	testing

### 3.3. Data acquisition process

**Fig 4:**
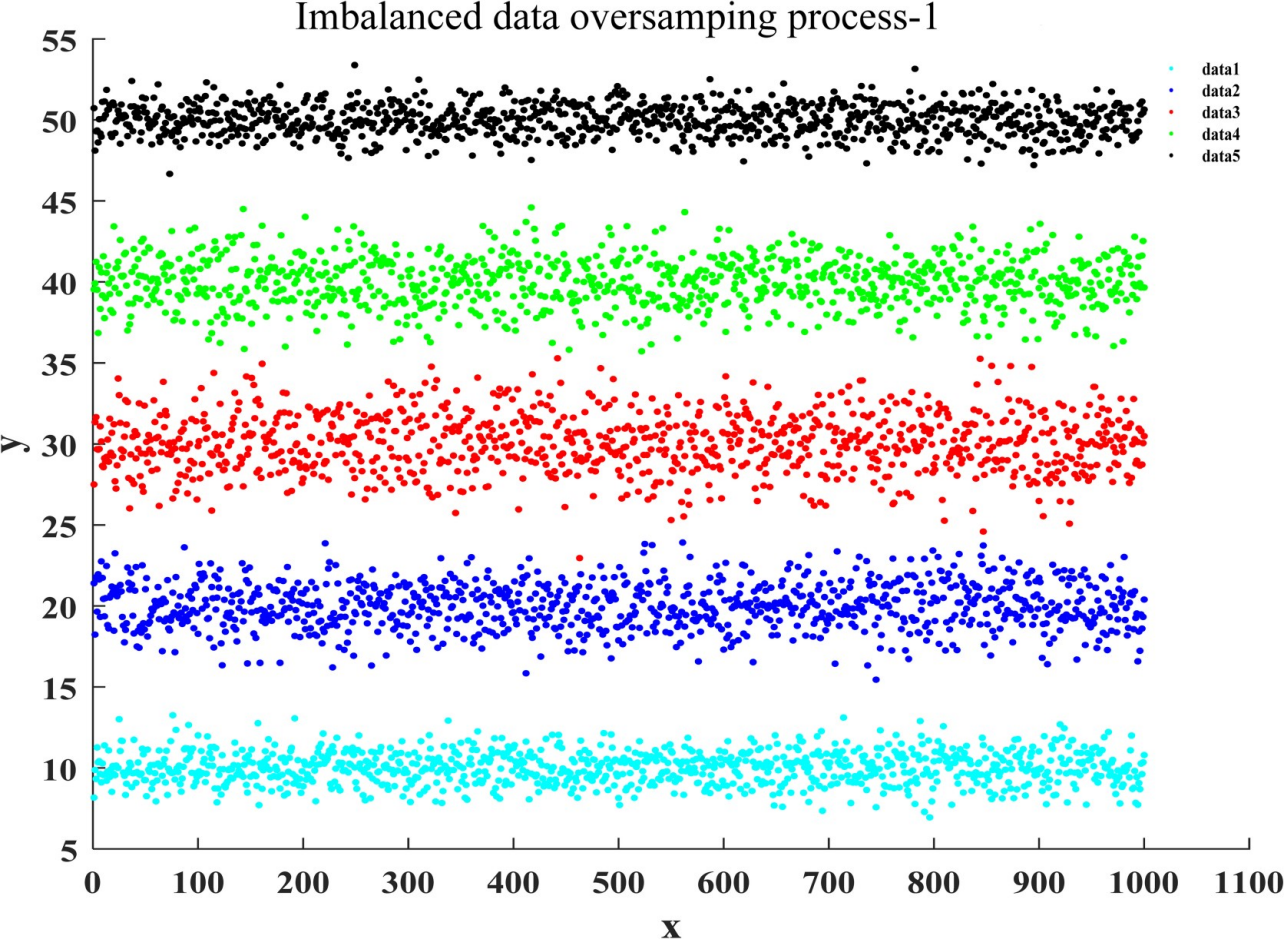
Data sorting of oversampling for imbalanced data.

**Fig 5:**
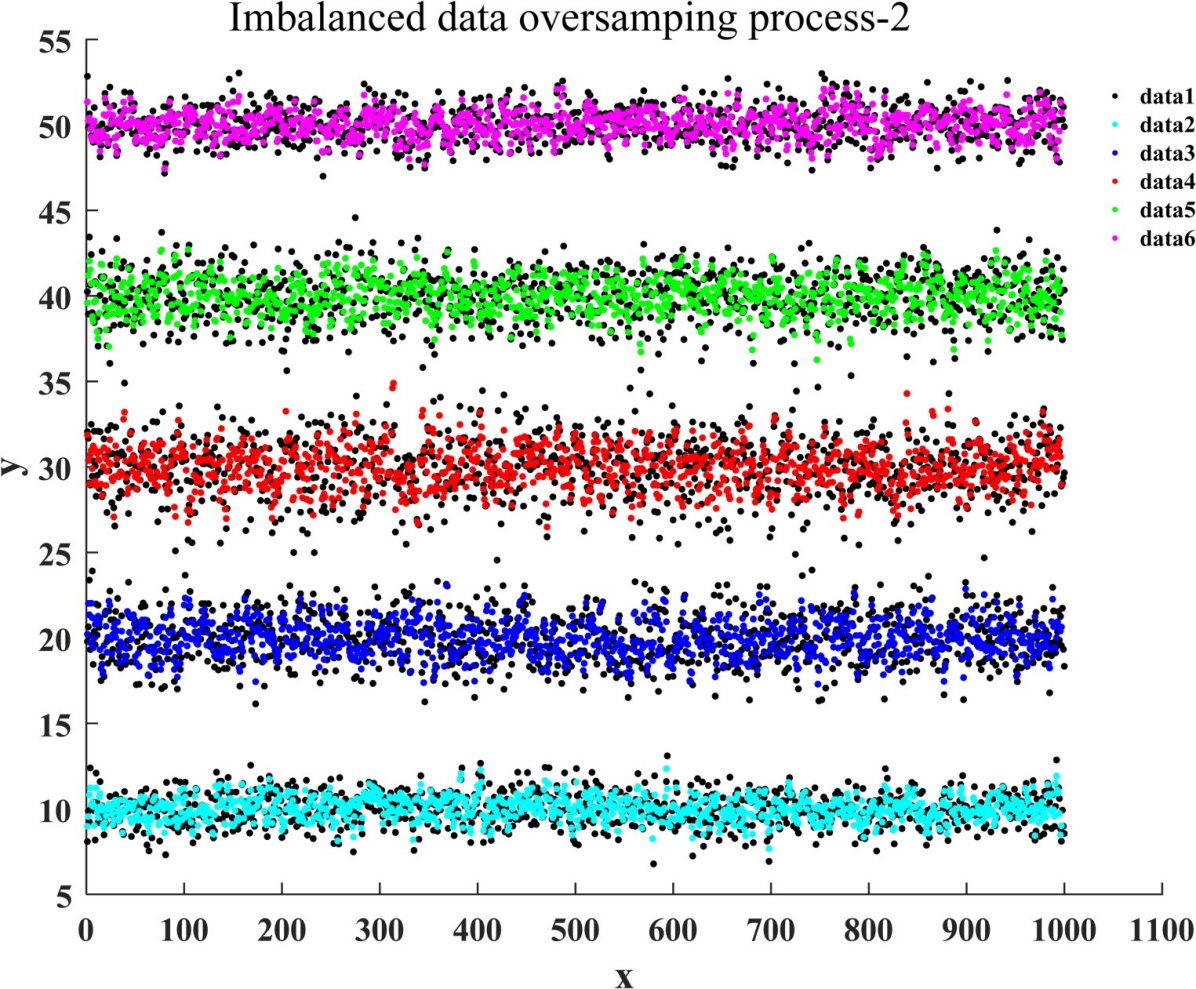
The 2-point sampling of oversampling for imbalanced data.

**Fig 6:**
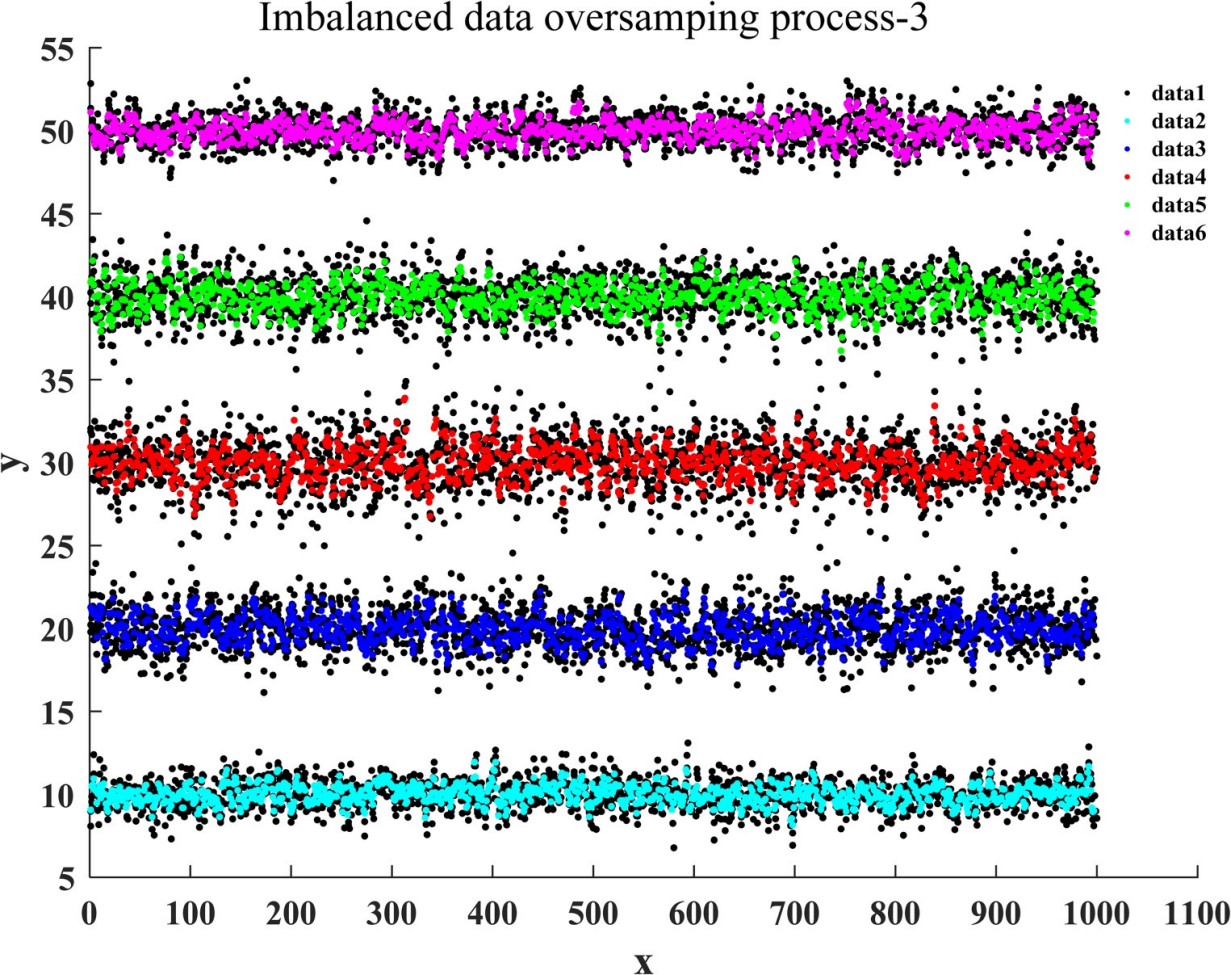
The 3-point sampling of oversampling for imbalanced data.

**Fig 7:**
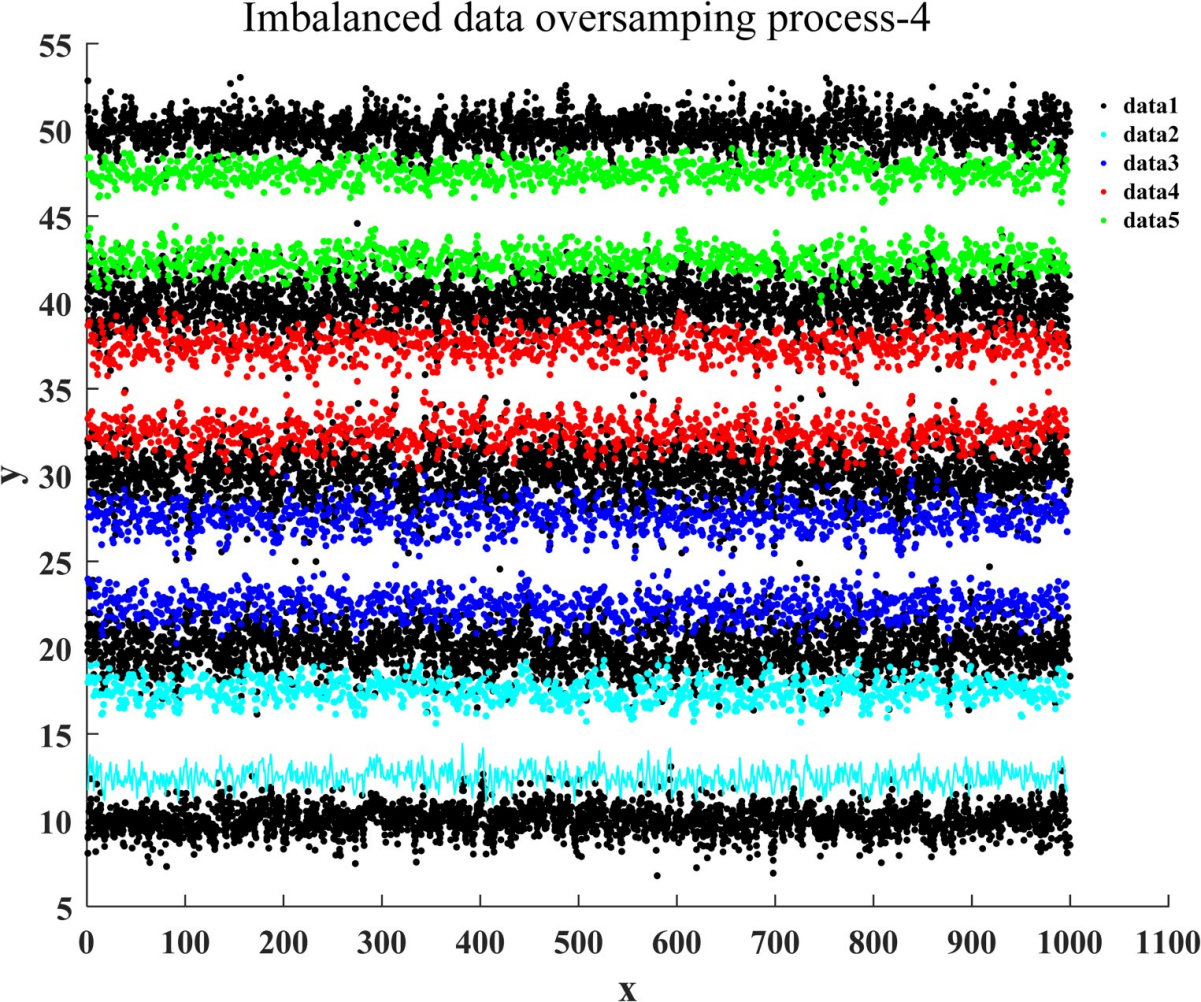
Inter-class sampling of oversampling for imbalanced data.

After the training of the proposed classification oversampling algorithm was passed, it was utilized to generate the sampled data for five categories of samples in market research. Figs 4–7 illustrate the complete oversampling process of this algorithm for imbalanced data, including four stages of data sorting by category, 2-point sampling within a class, 3-point sampling within a class, and inter-class sampling. The results show that the proposed algorithm has balanced the amount of data between minority class samples and majority class samples (Fig 7).

## 4. Algorithm evaluation

### 4.1 Single-indicator evaluation of algorithms

The performance of the data classification oversampling algorithm is mainly evaluated according to the degree of consistency between the predicted values of the sample classification and the actual values of the sample classification. Usually, the parameters defined in the confusion matrix are used to measure and evaluate the prediction accuracy of sample classification. Table 3 illustrates the composition of the confusion matrix. In the imbalanced data, the category of samples with a small amount of data was defined as a positive category and the category of samples with a large amount of data was defined as a negative category [34].

**Table 3:** Confusion matrix.

Category	Predicted positive category	Predicted negative category	True quantity
Actual positive category	True Positive (TP)	False Negative (FN)	TP+FN
Actual negative category	False Positive (FP)	True Negative (TN)	FP+TN
Forecasted total	TP+FP	FN+TN	

Furthermore, in Table 3, TP denotes that the predicted result is positive category, and is actually also the number of samples of the positive category. FN represents the number of samples predicted to be negative class, but in fact it is positive. Similarly, FP indicates that the predicted result is positive category, but is in fact the number of samples of the negative category. TN represents the number of samples predicted to be negative class, and is actually negative.

#### 4.1.1 Definition of single indicators

According to the definition of the confusion matrix prediction values in Table 3, four single evaluation indicators of the classification oversampling algorithm can be derived, including the accuracy ratio, precision ratio, specificity and recall ratio. Among them, the accuracy ratio is the proportion of the overall correct prediction of the sample, the precision rate is the proportion of the actual positive category data in the sample predicted to be the positive category, and the recall ratio is the proportion of the positive category samples identified among all the actual positive category samples. Specificity is the proportion of the negative category samples identified in all actual negative category samples. The calculation formulas for these four indicators are as following (see Eqs (7)–(10)).


$$Accuracy=\frac{TP+TN}{TP+TN+FN+FF}$$ (7)

Recall=TPTP+FN (8)

$$\mathrm{Pr}ecision=\frac{TP}{TP+FP}$$ (9)

$$Specificity=\frac{TN}{FP+TN}$$ (10)

#### 4.1.2 Evaluation and analysis of single indicators

After the proposed algorithm was trained on the training datasets, the performance of the proposed algorithm expressed in STCPS is verified by testing the dataset against the mainstream sampling algorithms including SMOTE, SVMOM, SMO+TLK and SVM+ENN [35–38]. In accordance with the statistical principles, when all data meet the test of reliability greater than 0.7 and validity greater than 0.6, the evaluation indicators’ values of the above algorithms are presented in Table 4.

**Table 4:** Single index evaluation of different algorithms.

Testing dataset	Algorithm	Recall	Precision	Specificity
**Market Research**	SMOTE	0.8934	0.8972	0.8986
	SVMOM	0.8731	0.9098	0.8893
	SMO+TLK	0.8857	0.9028	0.8922
	SVM+ENN	0.8943	0.8962	0.8998
	STCPS	0.9013	0.8937	0.8909

In Table 4, there is a negative correlation between the recall ratio and the precision ratio of data classification. The analysis shows that this is due to the small number of minority class samples in imbalanced data classification, which is prone to classification errors and leads to larger errors in the classification of TP samples and FN samples. Therefore, The single indicators method cannot accurately evaluate the performance of imbalanced data classification algorithms [39].

### 4.2 Comprehensive evaluation of the algorithm

#### 4.2.1 Selection of composite indicators

Through the comparative analysis of the single indicator evaluation results of different algorithms, it was found that single indicators were not applicable to the evaluation of classification oversampling algorithms for imbalanced data. For this reason, we used the composite indicators such as Accuracy, F-value and G-mean to evaluate the performance of classification algorithms as a whole in imbalanced data [40,41].

1. F-value

F-value is the harmonic average of both recall rate and precision ratio (see Eq (11)), which is closer to the smaller one of these two single evaluation indicator values. So, if the F-value is larger, then both the precision rate and the recall rate should be higher.


$$F=\frac{2\cdot Recall\cdot Precision}{Recall+Precision}$$ (11)

2. G-mean

G-mean is the square root of the multiplication of both recall ratio and specificity (see Eq (12)). If G-mean is larger, then both recall ratio and specificity should be larger.


$$G-mean=\sqrt{Recall\cdot Specificity}$$ (12)

3. AUC

AUC refers to the area enclosed by the ROC curve and the Horizontal and vertical axes (see Eq (13)), where the vertical coordinate TPR is the recall ratio and the horizontal coordinate FPR is 1-Specificity. If the AUC value is closer to the upper right corner, it means that the algorithm performance is better.


$$AUC=\int_{0}^{1}f(TPR\cdot FPR)dt$$ (13)

#### 4.2.2 Comprehensive evaluation of the algorithm

In the comprehensive evaluation experiment of the algorithm, the market research data was still used as the test dataset. After testing, the three composite indicators and their average values of the proposed algorithm and other mainstream sampling algorithms are obtained, as shown in Table 5.

**Table 5:** Comparison of composite indicators of different algorithms.

Testing dataset	Algorithm	G-mean	F-value	AUC	C I
**Market Research**	SMOTE	0.8960	0.8953	0.8028	0.8647
	SVMOM	0.8812	0.8911	0.7764	0.8496
	SMO+TLK	0.8889	0.8942	0.7902	0.8578
	SVM+ENN	0.8968	0.8952	0.8047	0.8657
	STCPS	0.8970	0.8975	0.8030	0.8655

### 4.3 Results and discussions

Through comparing Tables 4 and 5, it can be found that the magnitude of the composite indicator is related to the value of the single indicator, but the magnitude of the single indicators has less influence on the composite indicators. The experimental results show that it is difficult to evaluate the superiority of the algorithm by the level of single composite indicators such as AUC, F-value and G-mean. Finally, we took the average value of the three composite indicators (referred to as CI) as the final evaluation indicator. Moreover, through comparing the CI values of different algorithms, it is found that the classification oversampling method proposed in this paper does not show significant superiority in the composite indicator AUC compared with other algorithms, but the CI value of this algorithm is significantly higher than that of SMOTE, SVMOM and SMO+TLK algorithms, which indicates that this algorithm has good sampling functional capability for imbalanced data.

After the test simulation results of the five algorithms were compared (see Table 5), it can be seen that the G-mean, F-value and AUC values obtained from the proposed algorithm test are all greater than 0.8, especially the values of G-mean and F-value are close to 0.9. Therefore, it is deduced that both Recall and Specificity are approximated to 0.9. This indicates that the prediction rate of the proposed algorithm distinguishing between negative samples and positive samples is about 90%, and its accuracy is relatively high. In addition, when the AUC value is greater than 0.8, it indicates that both Recall and Precision values are greater than 0.8. This implies that most of the samples have been identified. The research results show that this algorithm has a high prediction coverage as well as a low error rate.

## 5. Conclusions

The classification oversampling method based on composite weights is proposed for multi-class imbalanced data. The algorithm first sorted the internal data of each class by the distance from the sample data to the hyperplane, and then calculated the data density around the sampling point. Furthermore, the original samples were sampled using the data sorting and data density as weights. Meanwhile, the sampled new data are assigned according to the information of the neighbors around the sampling point. After the designed algorithm is trained and tested, the new samples not only balance the number of the original samples, but also maintain the original data characteristics due to the consistency of their information assignment with the original sample data in general. Finally, the comprehensive evaluation method is used to compare the evaluation index of the proposed classification algorithm with other mainstream algorithms. The results demonstrate that the prediction accuracy of the positive and negative samples of this algorithm is about 90% which implies that it has a good recognition rate for positive and negative samples. The speed of the classification calculation in this study needs to be improved, due to the widespread applications of imbalanced data and the limited training samples of the improved algorithm. It is suggested that the next stage of the algorithm can be improved from data pre-processing. For imbalanced data samples, the classification oversampling algorithm based on composite weights has better effectiveness and generality, and is suitable for machine learning.
